# Tea Seed Kaempferol Triglycoside Attenuates LPS-Induced Systemic Inflammation and Ameliorates Cognitive Impairments in a Mouse Model

**DOI:** 10.3390/molecules27072055

**Published:** 2022-03-22

**Authors:** Tsung-Ming Yeh, Ching-Dong Chang, Shyh-Shyan Liu, Chi-I Chang, Wen-Ling Shih

**Affiliations:** 1 Department of Veterinary Medicine, National Pingtung University of Science and Technology, Pingtung 912, Taiwan; ytm@mail.npust.edu.tw (T.-M.Y.); cdchang@mail.npust.edu.tw (C.-D.C.); lad@mail.npust.edu.tw (S.-S.L.); 2 Department of Biological Science and Technology, National Pingtung University of Science and Technology, Pingtung 912, Taiwan; 3 General Research Service Center, National Pingtung University of Science and Technology, Pingtung 912, Taiwan

**Keywords:** flavonoid, LPS, ear, small intestine, Y-maze, human disease

## Abstract

(1) Background: The current research intended to obtain functional compounds from agricultural by-products. A functional tea seed flavonoid, kaempferol-3-O-[2-O-β-d-xylopyranosyl-6-O-α-L-rhanmopyranosyl]-β-d-glucopyranoside (KXRG), was isolated from tea seed dregs. We further determined its chemical structure and evaluated the protective effects of KXRG against local and systemic inflammation in vivo; (2) Methods: First, cytotoxicity and proinflammatory cytokine release were examined in a cell-culture system. The biological activities of KXRG were investigated in a mouse model of ear edema, and from inflammatory damage to organs as demonstrated by histologic examination, in addition to brain function evaluation using the Y-maze test. Serum biochemical analysis and western blotting were utilized to explore the related cellular factors; (3) Results: KXRG inhibited IL-6 in RAW264.7 cells at a non-toxic concentration. Further experiments confirmed that KXRG exerted a stronger effect than indomethacin in terms of the prevention of 12-O-tetradecanoylphorbol acetate (TPA)-induced ear inflammation in a mouse model. KXRG feeding significantly prevented LPS-induced small intestine, liver, and kidney inflammatory damage, as demonstrated by histologic examination. KXRG also significantly improved LPS-induced cognitive impairments. Serum biochemical analysis showed that KXRG elevated antioxidant capacity and reduced levels of proinflammatory cytokines. Western blotting revealed that KXRG reduced the COX-2 expression induced by LPS in mouse tissues; (4) Conclusions: KXRG can be purified from agricultural waste, and hence it is inexpensive, with large amounts of raw materials available. Thus, KXRG has strong potential for further development as a wide-use anti-systemic inflammation drug to prevent human disease.

## 1. Introduction

Flavonoids are a group of compounds with strong antioxidant activities that are widely present in many fruits and vegetables. They are polyphenolic plant secondary metabolites and are known as natural antioxidant compounds with a wide variety of beneficial health effects. Flavonoids have been shown to have anti-inflammatory and analgesic properties that help to maintain vascular and cerebrovascular functions. Thus, flavonoids help protect the peripheral and cerebral blood supply, which in turn slows down or prevents cardiovascular and cerebrovascular disorders [1]. As a decrease in cerebral blood flow is known to be an important contributor to cognitive decline; thus, increased blood flow to the brain is thought to result in better performance in terms of executive functioning and memory [2].

Lipopolysaccharides (LPS)-induced inflammation may lead to an immune response that causes tissue damage. Macrophages and dendritic cells are the key immune cells activated by LPS in damaged tissue that are involved in inflammation, a protective response of the host [3]. The expressions of proinflammatory mediators—including many cytokines, chemokines, and adhesion molecules—during the inflammatory process are regulated by the transcription factor NF-κB [4,5,6]. The anti-inflammatory effects of flavonoids are related to inhibition of the NF-κB pathway. The canonical NF-κB pathway is normally triggered by toll-like c receptors (TLRs), a specific microbial pattern recognition molecule, on immune cells. Stimulation of TLRs by their ligands, such as microbial product LPS, causes NF-κB activation and leads to tissue injury. Proinflammatory cytokines, such as TNF-α and IL-1, are key for activation of the NF-κB pathway, and are rapidly released upon tissue injury or infection [5].

Studies have shown that flavonoids modulate NF-κB signaling via inhibition of proinflammatory cytokines in cardiovascular diseases [7]. Natural flavonoids have been demonstrated to reduce the expression of inflammatory mediators associated with neuroinflammation, suggesting that they may prevent neurodegeneration or benefit patients with chronic central nervous system neuroinflammation [7,8,9]. Furthermore, it has been shown that several naturally occurring flavonoids inhibit LPS-induced inflammation through blocking of NF-κB activation and inducible nitric oxide synthesis (iNOS) expression [10]. Therefore, flavonoids have recently attracted much attention in light of their potential beneficial effects in disorders associated with inflammation.

The tea plant (*Camellia brevistyla* (Hay.) Coh.-Stuart.) is native to south-east Asia, including Taiwan. Flavonoids are abundant in the Camellia genus within the Theaceae family. Studies have suggested that the consumption of tea provides protection against inflammatory diseases due to tea being rich in anti-inflammatory components [11,12]. Both tea leaves and oils from Camellia species have been demonstrated to possess antioxidant and anti-inflammatory properties [13]. The majority of previous studies have been focused on extracts from leaves, while recent studies have indicated that flavonoids extracted from seeds also show promising results. For example, flavonoids from seeds of *C. oleifera* (Abel.) have been demonstrated to possess great anti-inflammatory activity in terms of controlling NO production in LPS-stimulated macrophages [14], while flavonoids from *C. sinensis* seeds were found to ameliorate TNF-α-induced insulin resistance in a human liver cancer cell line [15]. Oils from seeds of *C. brevistyla* were found to exert a protective effect against indomethacin-induced gastrointestinal mucosal damage [16].

Edible oils are often extracted from seeds of native *C. brevistyla*, which contain high levels of unsaturated fatty acids [17]. The seed dregs are of low economic value and are normally treated as agricultural waste. Ethanol extracts from *C. brevistyla* seed dregs have been shown to reduce blood pressure in a mouse model, suggesting that these extracts have antihypertensive properties and may reduce the risk of cardiovascular diseases [18]. However, no study has isolated a single compound from a seed extract and explored the mechanisms of its bioactivities. Therefore, in this study, we isolated a bioflavonoid, kaempferol-3-O-[2-O-β-d-xylopyranosyl-6-O-α-*L*-rhanmopyranosyl]-β-d-glucopyranoside (KXRG), from defatted seeds of *C. brevistyla*, and investigated its anti-inflammatory effects and impact on cognitive impairments.

## 2. Results

### 2.1. Structural Identification of KXRG

**Figure 1:**
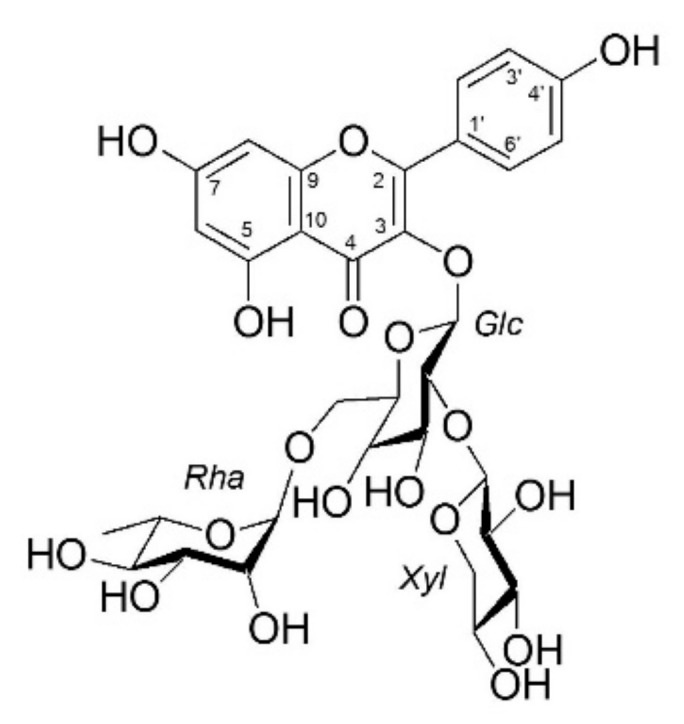
The chemical structure of KXRG isolated from *C. brevistyla* seeds. Nuclear magnetic res-onance (^1^H NMR and ^13^C NMR) techniques were used for the structural determination of KXRG. NMR spectra were recorded on a Varian spectrometer (400 MHz for ^1^H NMR and 100 MHz for ^13^C NMR) in CD_3_OD.

The structure of isolated tea seed bioflavonoid, kaempferol-3-O-[2-O-β-d-xylopyranosyl-6-O-alpha-L-rhanmopyranosyl]-β-d-glucopyranoside (KXRG), is shown in Figure 1.

### 2.2. Anti-Inflammatory Activity of KXRG in Cultured RAW264.7 Cells

**Figure 2:**
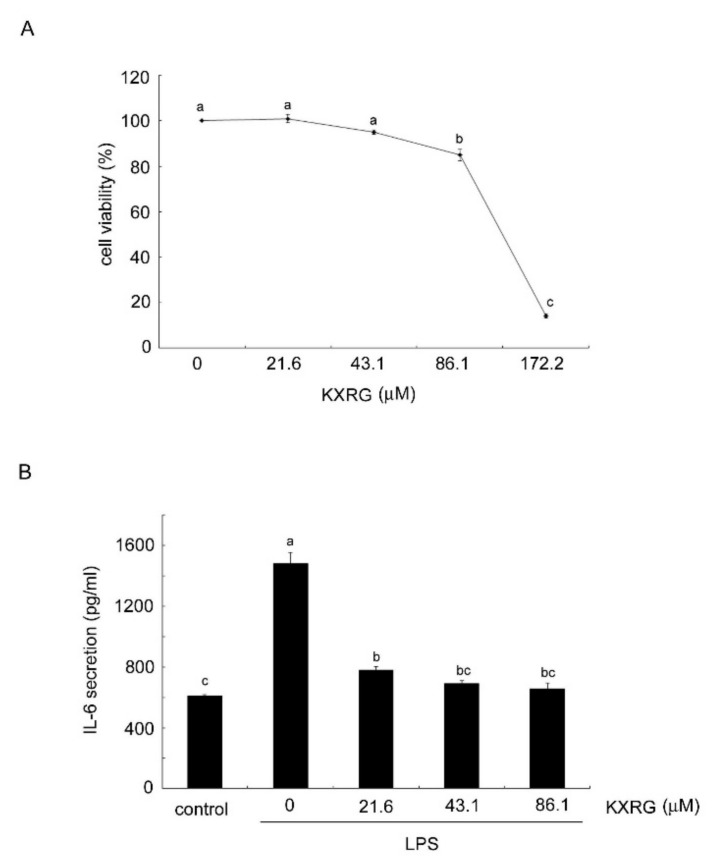
Anti-inflammatory activity of KXRG in cultured RAW264.7 cells. (**A**) Cytotoxicity of KXRG towards RAW264.7 cells, (**B**) effect of KXRG on the inhibition of IL-6 secretion in LPS-stimulated RAW264.7 cells. Cells were pre-treated with non-toxic concentrations of KXRG for 2 h, followed by incubation with 1 µg/mL LPS. IL-6 levels in the culture media were measured. Bars marked with different letters indicate statistically significant differences among groups at *p* < 0.05.

The results of a cytotoxicity assay are shown in Figure 2A, in which cells were treated with KXRG for 24 h. Significant cytotoxicity occurred at concentrations above 62.5 μg/mL, at which cell survival was approximately 80%. Subsequent experiments were performed to measure the level of secreted proinflammatory cytokine IL-6. As shown in Figure 2B, KXRG inhibited LPS-induced IL-6 secretion at a non-cytotoxic concentration in a dose-dependent manner. However, in our system, LPS-induced TNF-α was not suppressed by KXRG as expected (data not shown).

### 2.3. KXRG Reduced TPA-Induced Local Inflammation

**Figure 3:**
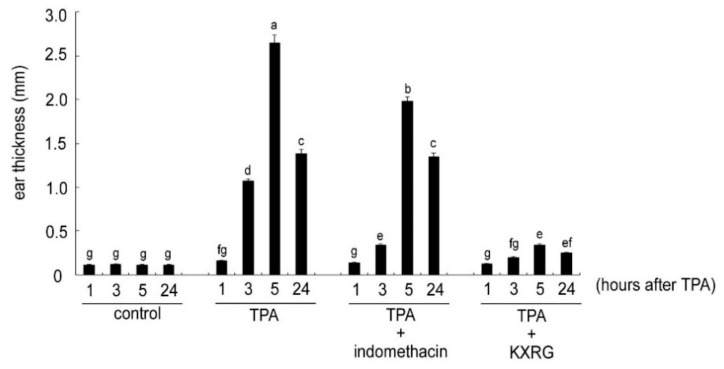
Effect of KXRG on the inhibition of 12-O-tetradecanoylphorbol acetate (TPA)-induced mouse ear edema in Balb/c mice. After treatment with KXRG (0.5 mg) or indomethacin (0.5 mg) for 30 min, 2 μg of TPA were applied to both the inner and outer ear surfaces. At 1, 3, 5, and 24 h after TPA administration, the ear thickness was measured. Bars marked with different letters indicate statistically significant differences among groups at *p* < 0.05.

The possible topical anti-inflammatory role of KXRG on skin was examined using an acute ear edema mouse model, in which edema was induced by TPA application in Balb/c mice [19]. As shown in Figure 3, the skin thickness started to increase significantly at 3 h after TPA treatment, and reached a maximum at 5 h, which lasted for at least 24 h. Indomethacin pretreatment significantly reduced the TPA-mediated ear edema by 26%. Compared with the same dose of indomethacin, KXRG prevented ear edema more efficiently than indomethacin, resulting in an 84.6% inhibition.

### 2.4. KXRG Prevented LPS-Induced Systemic Inflammation

**Figure 4:**
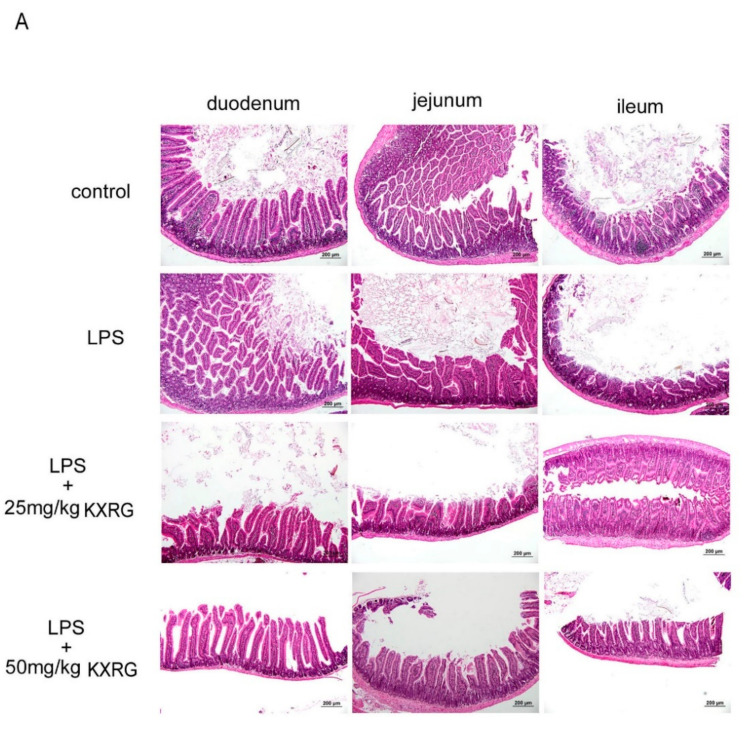

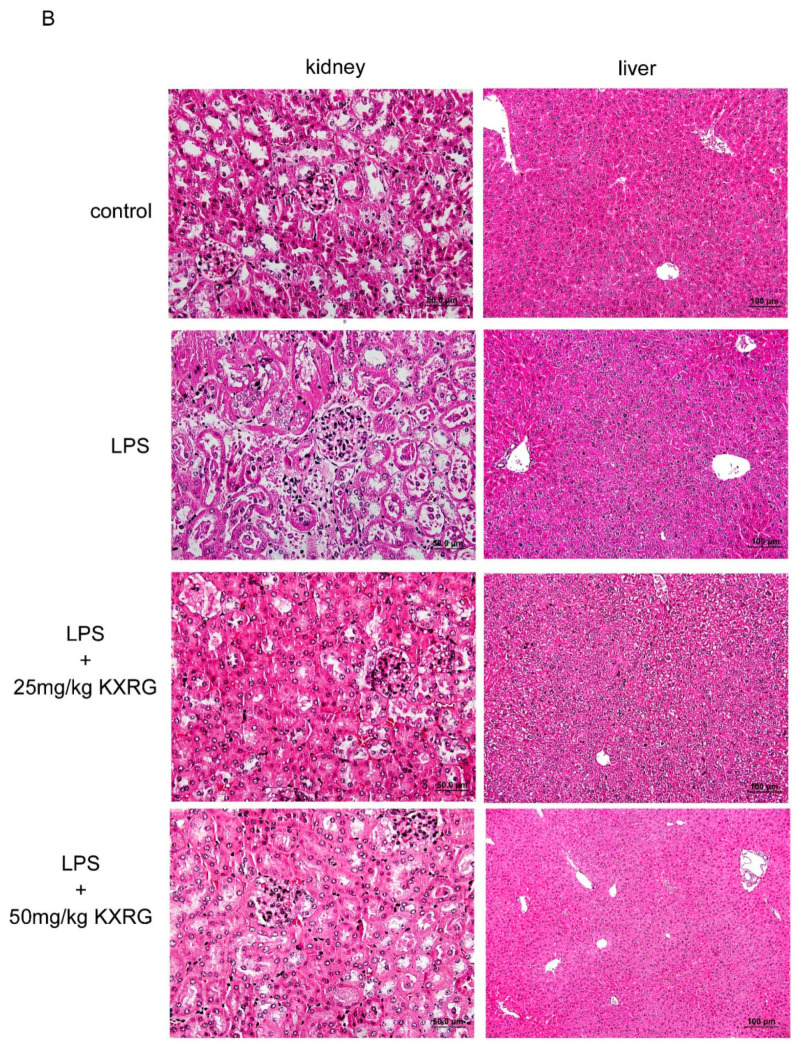

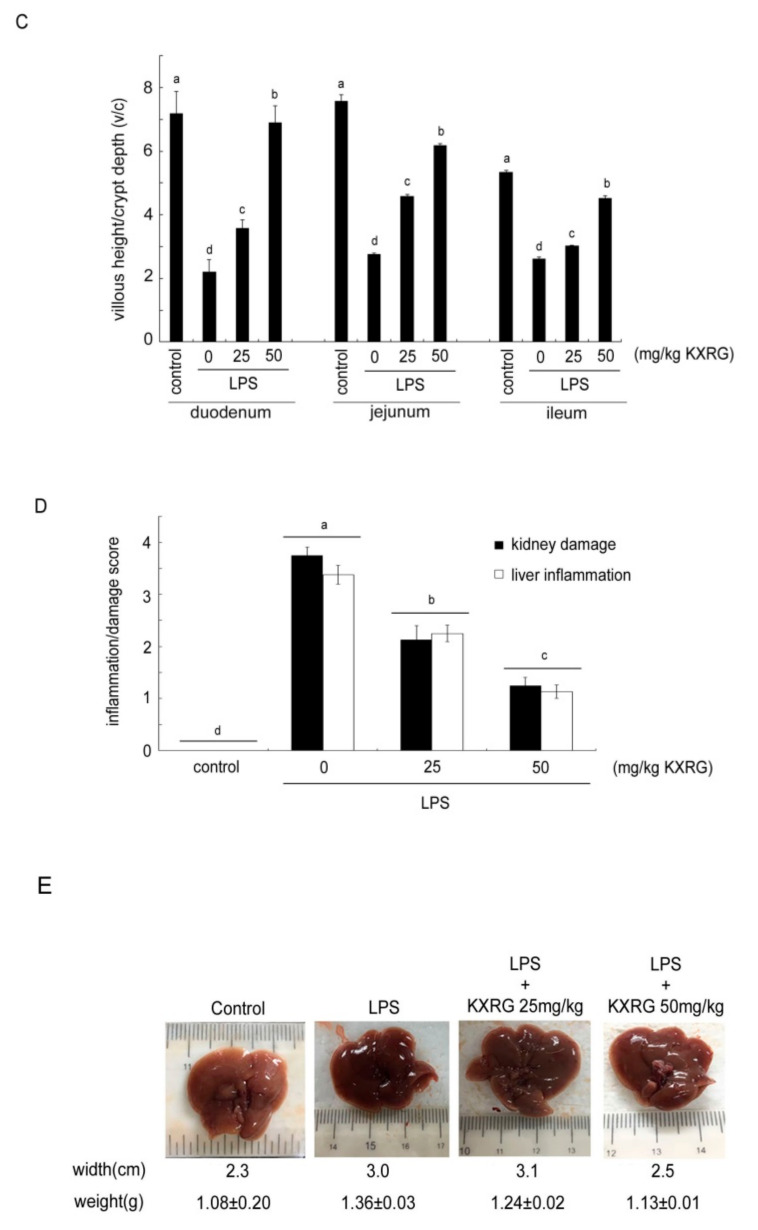
KXRG prevents LPS-induced systemic inflammation. Morphological changes in (**A**) the small intestine and (**B**) the kidneys and liver in controls and mice fed with 25 mg/kg or 50 mg/kg KXRG followed by LPS challenge. (**C**) Villous height/crypt depth in the duodenum, jejunum, and ileum of the different treatment groups. (**D**) Liver inflammation and kidney damage scores. (**E**) Liver weight of the mice with LPS treatment. Bars marked with different letters indicate statistically significant differences among groups at *p* < 0.05.

LPS intraperitoneal injection elicits systemic acute inflammation that affects many organs, and is a well-established C57BL/6 mouse model [20]. The acute injuries are severe, especially in the small and large intestine, liver, and kidneys [20,21,22]. To further evaluate the anti-inflammatory activity of KXRG in terms of tissue protection in the whole organism, mice were fed KXRG at 25 mg/kg or 50 mg/kg then challenged with LPS. LPS caused obvious small-intestine epithelial shedding, villi fracture, mucosal atrophy, and fusion of adjacent villi, and the villus had shortened (Figure 4A, as compared with the control group and LPS-treated group). In the two KXRG-fed groups, especially in the group fed a high dose, the morphology of the villus was more intact than in the mice treated with with LPS alone (Figure 4A, as compared with the LPS-treated group and the KXRG combined with LPS-treated group). The villus height (VH) and crypt depth (CH) indicate the absorptive function of the intestine, and were therefore measured in the mice. Figure 4C illustrates the lower VH and decreased V/C ratio in the duodenum, jejunum, and ileum in the LPS-treated mice as compared with the control mice. KXRG feeding protected the intestinal villi from inflammation and damage very effectively. Additionally, LPS induced obvious acute kidney injury, as demonstrated by aberrant epithelial cells, interstitial edema, and renal tubule dilation (Figure 4B). LPS also induced septic liver injury, as shown in Figure 4B. KXRG feeding significantly protected the kidneys and liver from inflammatory-related damage according to histologic examination (Figure 4B) and scoring (Figure 4D). Visual observation of the liver highlighted inflammation and congestion with swelling, in addition to increases in weight and volume, in the LPS-injected mice as compared with the control mice. However, the livers of the KXRG-fed mice displayed an appearance similar to that of the normal mice in terms of color, weight, and size, especially in the high-dose KXRG-fed mice (Figure 4E). These results showed that tea seed flavonoid KXRG has significant beneficial effects against symptoms of systemic inflammation.

### 2.5. KXRG Attenuated LPS-Induced Brain Cognitive Impairments

**Figure 5:**
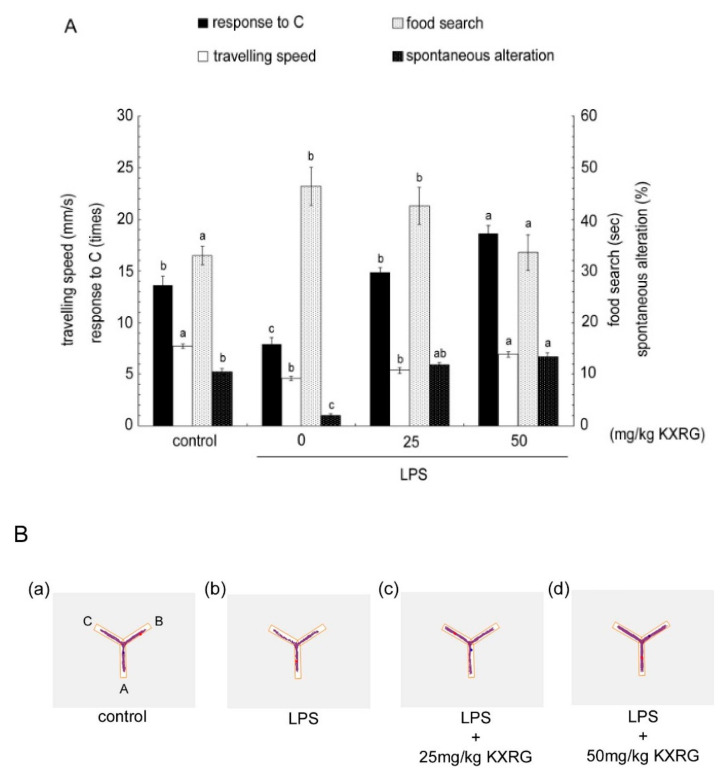

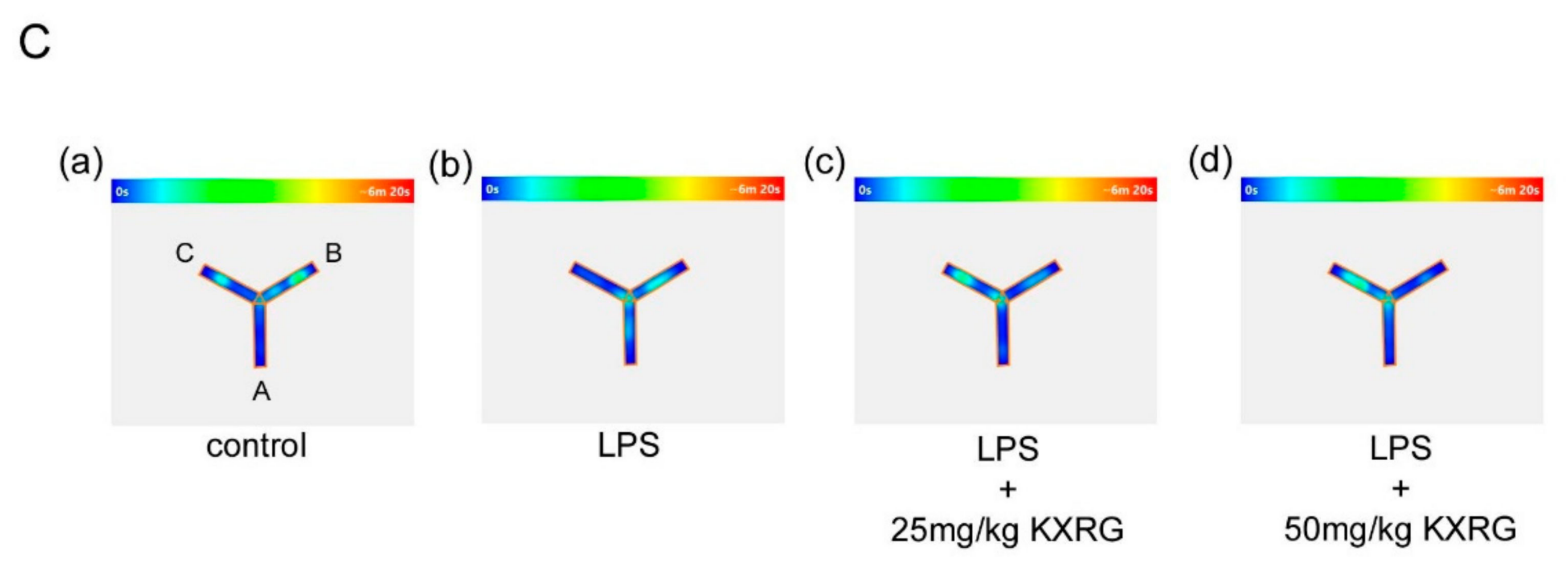
Effects of KXRG on brain cognitive impairments in mice with or without LPS treatment. (**A**) The *y*-axes indicate the different functional evaluations. Bars marked with different letters indicate statistically significant differences among groups at *p* < 0.05. (**B**) Mouse movement trajectory within 10 min. (**C**) Time spent by mice in each zone (**A**–**C**) within 10 min. Colors as shown at the top of the image. Time increases from blue to red.

The Y-maze is a convenient behavioral test by which to evaluate cognition and spatial memory. As shown in Figure 5A, compared with the control mice, the LPS-treated mice lost a natural desire to explore new environments, as the mice entered the C arm of the maze fewer times; they also presented with reduced mobile activity, spent more time looking for food, and exhibited poor memory and cognition, as reflected by less spontaneous alteration behavior. The mice fed with KXRG exhibited a greater desire to explore a new environment and had longer stays in arm C in the Y-maze test, in addition to a greater speed of movement and a higher percentage of direction alteration behavior. Furthermore, using the Y-maze with food as a reward, the short-term spatial memory in the different groups of mice was compared. As shown in Figure 5A, the LPS-treated mice required a greater amount of time to find the food than the normal mice, indicating that LPS injection caused deterioration in cognitive memory. All the mice in the treatment groups with KXRG feeding exhibited improvement in short-term spatial memory, especially the mice fed a higher dosage. Figure 5B shows the path of each group of mice during the 10-min exploration, from which it can be seen that the trajectory of the LPS-treated mice was shorter than that of the control group. KXRG feeding rendered the mice more mobile. Figure 5C shows the residence time in color, with time increasing from blue to red. The results showed clearly that the LPS-treated mice stayed in zone C for a significantly shorter duration, while the mice in the KXRG-fed groups spent a significantly greater amount of time in the C-terminus.

### 2.6. KXRG Strengthened the Antioxidant Function and Reduced Proinflammatory Cytokines in Mice

**Figure 6:**
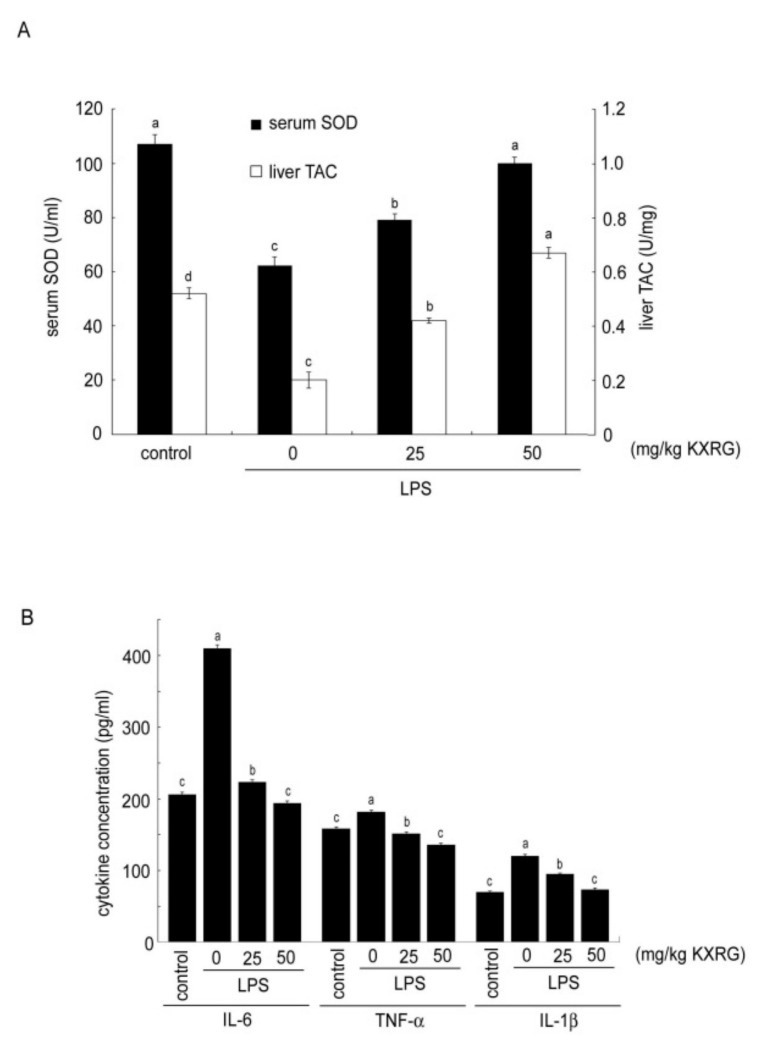
Effects of KXRG in mice with or without LPS treatment on (**A**) serum SOD activity and liver TAC, (**B**) cytokines IL-6, TNF-α, and IL-1β in the serum. Bars marked with different letters indicate statistically significant differences among groups at *p* < 0.05.

SOD is one of the critical antioxidant enzymes that provide a defensive mechanism against free radical-induced tissue damage. In addition to the enzymatic antioxidant machinery, the non-enzymatic antioxidant capacity was measured according to the Trolox equivalent antioxidant capacity (TAC). As shown in Figure 6A, KXRG reversed the LPS-induced serum SOD activity and liver EAC reduction significantly, with a higher dose of KXRG eliciting a stronger effect than a lower dose. Cytokines possess multiple functions and play critical roles in host defense. LPS upregulation of various proinflammatory cytokines in mice is a powerful model by which to evaluate anti-inflammatory compounds in vivo. As shown in Figure 6B, IL-6, TNF-α, and IL-1β were markedly increased in the LPS-treated mice as compared with the control mice, which confirmed that LPS injection elicited systemic inflammatory effects. Oral administration of KXRG prevented increases in these three cytokines in response to LPS, and the inhibition effect of higher-dose KXRG feeding was better than that observed with a lower dose.

### 2.7. KXRG Reduced the LPS-Induced COX-2 Expression in Mouse Tissue

**Figure 7:**
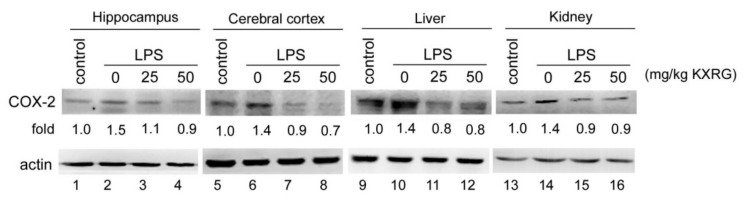
Western blotting analysis of COX-2 expression in the brain, liver, and kidneys. Equal amounts of protein lysates of the hippocampus, cerebral cortex, liver, and kidneys were prepared from LPS-injected mice with or without KXRG feeding. The COX-2 expression level is shown as the fold change normalized against actin and compared with the control group.

Specific organs were collected in our mouse model, and the expression of cyclooxygenase-2 (COX-2)—which is responsible for the production of prostaglandins that mediate the inflammatory process and related symptoms—was measured. As shown in Figure 7, COX-2 enzyme expression was significantly induced in the LPS-injected mice at a level of about 1.4- to 1.5-fold that of the control mice. In the KXRG-fed mice, the COX-2 expression levels in examined tissue were downregulated, with expressions similar to those of the control group.

## 3. Discussion

In this study, we examined the anti-inflammatory activities of KXRG on LPS-stimulated RAW264.7 cells, TPA-induced local inflammation, and LPS-induced systemic inflammation in mice. The results of our in vitro study demonstrated that KXRG exhibits anti-inflammatory properties, quashing IL-6 production in LPS-stimulated macrophages. In our animal model, KXRG inhibited the development of ear edema in TPA-treated mice and reduced the organ damage caused by LPS intraperitoneal injection. Additionally, KXRG reduced cognitive impairments, improved antioxidant function, and alleviated the production of proinflammatory cytokines caused by LPS injection. Our results indicated that KXRG isolated from seed dregs of *C. brevistyla* is a bioactive flavonoid that has potential beneficial effects against inflammation.

Kaempferol and its glycosides have been demonstrated to possess anti-inflammatory activities via in vitro and in vivo studies [23]. Generally, the poor water solubility of flavonoids restricts their use; however, KXRG is a kaempferol triglycoside compound, which exhibits a better water solubility than its aglycone, kaempferol. After oral administration, KXRG is absorbed through the intestinal cells and hydrolyzed by biological hydrolases into aglycone, which increases its bioavailability [24]. Macrophages are crucial immune cells in the immune defense system of the body, and their activation releases a variety of inflammatory cytokines and mediators. In vitro models using LPS-stimulated RAW264.7 macrophages have been widely used to assess the anti-inflammatory effects of many natural products, as LPS is an effective inducer of the inflammatory response in macrophages. In this study, we first employed this model to identify non-cytotoxic doses of KXRG, and confirmed that KXRG inhibited the expression of IL-6 in macrophages. Subsequently, using in vivo mouse models, we found that KXRG exerted a significantly stronger effect in terms of alleviating ear edema than indomethacin when applied at the same concentration.

Intraperitoneal injection of LPS can induce pathological intestinal epithelial cell apoptosis and cell shedding, which are important processes in systemic and intestinal inflammatory diseases [25]. We found that KXRG feeding prevented small-intestine epithelial shedding and mucous damage, as well as kidney damage and liver inflammation, caused by LPS (Figure 4A,B). To further investigate the effects of KXRG, we administered KXRG at 25 and 50 mg/kg and evaluated its protective effects by scoring the tissue damage to intestinal villi of different regions, the kidneys and the liver (Figure 4C–E). The results showed that tea seed flavonoid KXRG has an excellent protective effect against organ damage, suggesting that KXRG at 25–50 mg/kg exerts significant beneficial effects against systemic inflammation caused by LPS. Some evaluation indicators were more significant at a dose of 50 mg/kg, the main reason for which is likely the differences in the biochemical metabolism and physiological pathways of different indicators.

Our results revealed that KXRG selectively inhibits COX-2 over iNOS, and IL-6 over TNF-α. Although the mechanisms of action and cellular targets are as yet unclear, the results were sufficient to evidence an anti-inflammatory effect of KXRG in mice.

Tsoyi et al. reported that the IFN-β/JAK2/STAT1 signaling pathway is involved in carbon monoxide-releasing molecule 2 (CORM-2)-mediated iNOS gene expression, but not that of COX-2. Although most studies have shown that LPS can strongly activate NF-κB transcription factor and downstream iNOS and COX-2, in the system of Tsoyi et al., inhibition of NF-κB by CORM-2 appeared not to be responsible for the distinct regulation of iNOS and COX-2. The researchers suggested that inhibition of iNOS relies on both NF-κB and STAT1, rather than transcription factors CCAAT enhancer binding protein (C-EBP), NF-κB, and AP-1 [26,27]. Cho et al. previously performed testing of a series of chrysin (a natural flavone compound) derivatives, and found that only 5,7-diacetylflavone (Ch-4) derivatives were particularly effective against COX-2. The inhibitory effects of different derivatives on NO act to different degrees [28]. Differential regulation of iNOS and COX-2 expressions has also been demonstrated in another experimental system [29].

Although IL-6 and TNF-α have been reported to be involved in various aspects of the inflammation process [30], continuing research has shown that these two cytokines have multiple biological functions and complex molecular regulation [31]. Previous study suggested contributions of protein kinase C (PKC) and adenylate cyclase to IL-6 induction [32]. Potential transcriptional regulatory elements—such as glucocorticoid responsive element (GRE), AP-1, c-fos serum responsiveness element homology, and NF-κB binding sites—were identified within the IL-6 promoter region [33]. In the TNF gene, NF-κB, AP-1, and Sp-1 binding sequences have been found [34]. However, the regulatory effects on and roles of these motifs in TNF-α expression are not known.

Taking this information together, the pleiotropy, redundancy, and networks of these inflammatory molecules are more complex than thought. At present, our evidence tends to support that KXRG might act via specific pathway or target, and it is more inclined to inhibit COX-2 and IL-6.

LPS has been used to experimentally induce memory impairments and neuroinflammatory responses [22,35]. We employed the Y-maze test to assess the effects of KXRG feeding on LPS-induced memory impairments in mice [36]. The results showed that daily administration of KXRG (25 and 50 mg/kg) improved LPS-induced memory impairments, including improvement in the desire to explore new environments, greater mobile activity, reduced duration spent looking for food, improved memory, and improved cognition (Figure 5). Therefore, KXRG exerts activity against inflammation in terms of improving cognition and short-term memory, which are related to enhancement of the motivation and desire of the mice to explore new environments.

Spatial learning and memory are known to require consolidative coordination from the hippocampus [37]. Injection of LPS stimulates the immune system to produce proinflammatory cytokines—such as IL-1α, IL-1β, IL-6, and TNF-α—in the mouse hippocampus and cortex [38]. Therefore, we investigated whether KXRG suppressed LPS-induced increases in the levels of various proinflammatory cytokines in the serum and the protein expression of COX-2 in organs, including the hippocampus and cortex. The results showed that oral administration of KXRG inhibited LPS-induced IL-6, TNF-α, and IL-1β increases (Figure 6B), which are the key cytokines in inflammatory diseases [4], and prevented increases in inflammatory enzyme COX-2 in the brain, liver, and kidneys. When macrophages are activated by LPS, two key proinflammatory enzymes, COX-2 and iNOS, are often expressed in inflamed tissue. Although both are involved in the symptoms of inflammation, the regulation of their expression differs. Many non-steroidal anti-inflammatory drugs (NSAIDs) are selective inhibitors against COX-2, and the iNOS enzyme itself can be a useful therapeutic target. Thus, inhibition of COX-2 can provide relief from symptoms of inflammation and pain. In our study, the dramatic increase in COX-2 production in various tissues following stimulation by LPS was sharply attenuated by KXRG (Figure 7), indicating its strong anti-inflammatory potential [39].

LPS may provoke extensive organ damage, including liver damage. Liver damage induced by LPS generally indicates disruption to the liver cell metabolism, which leads to characteristic changes in serum enzyme activities. The process is known to be associated with increased release of reactive oxygen intermediates [40,41] and a resultant rise in lipid peroxidation [42]. Anti-oxidants from natural sources have been proven to be beneficial in reversing the hepatotoxicity and oxidative stress produced by LPS or toxicity from drugs [43]. Several flavonoids have been reported to have hepatoprotective effects [44,45,46]. Our results showed that KXRG reversed LPS-induced liver damage, which was confirmed by biochemical assays of serum SOD activity and the liver TAC. SOD is an important antioxidant enzyme that acts as the first line of defense in cells. Equilibrium of the enzyme to normal physiological conditions will help organs to overcome chronic oxidative stress. In addition to the enzymatic antioxidant machinery, we demonstrated that the non-enzymatic antioxidant capacity as measured by the TAC could be restored by KXRG (Figure 6A).

## 4. Materials and Methods

### 4.1. Plant Material

The tea tree plant (*Camellia brevistyla* (Hay.) Coh.-Stuart.) was cultivated in Lugu village, Nantou County, Taiwan. The plant material was identified by Prof. Sheng-Zehn Yang, Department of Forestry, National Pingtung University of Science and Technology. A voucher specimen has been deposited in the Graduate Institute of Biotechnology, National Pingtung University of Science and Technology. Defatted tea-seed meal of *C. brevistyla* was purchased from a farmer (batch number NCB-19-S01).

### 4.2. Extraction, Isolation, and Identification of Kaempferol-3-O-[2-O-β-d-xylopyranosyl-6-O-α-L-rhanmopyranosyl]-β-d-glucopyranoside (KXRG)

Defatted tea-seed meal was dried in an oven at 60 °C until it was of constant weight, and then processed into powder, sieving through a No. 20 mesh. The powdered sample of 1 kg was extracted three times with 95% ethanol at 60°C for two hours. The combined ethanol extract was evaporated in a vacuum to generate a blank residue (115 g), which was suspended in H_2_O (1000 mL) then partitioned sequentially using EtOAc and n-BuOH (1000 mL × 3) to obtain EtOAc (3.0 g), n-BuOH (82.1 g) and H_2_O (21.9 g) fractions. The n-BuOH fraction was chromatographed on an open Diaion HP-20 column (6 × 60 cm) and eluted with mixtures of water and methanol of reducing polarity to afford 11 fractions as follows: fr. 1 (2500 mL, water), fr. 2 (2500 mL, water-methanol (9:1)), fr. 3 (2000 mL, water-methanol (8:2)), fr. 4 (2000 mL, water-methanol (7:3)), fr. 5 (2000 mL, water-methanol (6:4)), fr. 6 (2000 mL, water-methanol (5:5)), fr. 7 (2000 mL, water-methanol (4:6)), fr. 8 (2000 mL, water-methanol (3:7)), fr. 9 (2000 mL, water-methanol (2:8)), fr. 10 ((3000 mL, water-methanol (1:9)), and fr. 11 (4000 mL, methanol). Fractions 4 and 5 (4.2 g) were combined and further chromatographed on an open RP-18 column (3 × 45 cm), eluted with water-methanol (8:2 to 0:1) to obtain six fractions (each 500 mL), fr. 4A-fr. 4F. HPLC of fr. 4C (210 mg) on a Phenomenex Luna C-18 column eluted with water-acetonitrile (9:1 to 1:1), 1 mL/min, yielded 1 (18.1 mg, tR = 16.1 min). Compound 1 was identified as kaempferol-3-O-[2-O-β-d-xylopyranosyl-6-O-alpha-L-rhanmopyranosyl]-β-d-glucopyranoside (KXRG): yellow amorphous solid; [α]25D–55.2 (c = 0.6, MeOH); UV (MeOH) λmax (log ε) nm: 268 (4.10), 318 (4.30), 354 (4.12, sh); IR (KBr) cm^−l^: 3316, 1693, 1658, 1595, 1510, 1070, 832; ESI-MS (Agilent, Inc., Santa Clara CA, USA)*m*/*z* 749 [M + Na]^+^; ^1^H-NMR (Agilent, Inc., Santa Clara, CA, USA) (400 MHz, CD3OD) δH 8.02 (2 H, d, *J* = 8.8 Hz, H-2′, H-6′), 6.88 (2 H, d, *J* = 8.8 Hz, H-3′,H-5′), 6.35 (l H, d, *J* = 1.5 Hz, H-8), 6.15 (l H, *J* = 1.5 Hz, H-6), 5.37 (l H, d, *J* = 7.2 Hz, Glc H-l), 4.78 (l H, d, *J* = 6.8 Hz, Xyl H-l), 4.49 (l H, d, *J* = 1.5 Hz, Rha H-l), 4.0–3.2 (15 H, m), l.08 (3 H, d, *J* = 6.0 Hz, Rha H-6); 13C-NMR (CD3OD, 75 MHz): δC 179.3 (C-4), 165.6 (C-9), 163.1 (C-7), 161.1 (C-5), 158.8 (C-2), 158.2 (C-4‘), 134.7 (C-3), 132.3 (C-2′, 6′), 122.8 (C-1′), 116.1 (C-3′, 5′), 105.6 (C-10), 105.0 (C-1 Xyl), 102.0 (C-1 Rha), 100.8 (C-1 Glu), 99.9 (C-6), 94.9 (C-8), 81.7 (C-2 Glu), 78.0 (C-3 Glu), 76.8 (C-3 Xyl), 76.7 (C-5 Glu), 74.6 (C-2 Xyl), 73.7 (C-3 Rha), 72.1 (C-2 Rha), 72.0 (C-4 Rha), 71.2 (C-4 Glu), 70.9 (C-4 Xyl), 69.6 (C-5 Rha), 68.0 (C-6 Glu), 66.5 (C-5 Xyl), 17.85 (C-6 Rha). The purity of KXRG was determined to be greater than 98% by HPLC-UV (Hitachi, Inc., Tokyo, Japan) analysis. The NMR spectrum, IR spectrum, Mass spectrum, HPLC chromatogram and purity calculation were presented in Supplementary Materials.

### 4.3. Chemicals and Reagents

The chemicals used in the extraction and Western blotting procedures, as well as MTT and LPS, were all purchased from Sigma (St. Louis, MO, USA). Cell culture media and antibiotics were purchased from Gibco BRL (Grand Island, NY, USA).

### 4.4. RAW264.7 Cell Culture, Cytotoxicity, and Treatment

Murine RAW264.7 macrophage cells were kindly provided by Dr. Tzou-Chi Huang of our university. RAW264.7 cells were maintained in Dulbecco’s modified Eagle medium (DMEM) containing 10% fetal bovine serum, 2 mM glutamine, 100 U/mL of penicillin and 100 μg/mL of streptomycin in a 37 °C incubator with 5% CO_2_. For the experiments, cells were detached by pipetting, centrifuged, and plated in fresh medium. Cytotoxicity analysis of KXRG evaluated by MTT assay was performed according to our previous work [47]. Various non-toxic concentrations of KXRG were added to RAW264.7 cells at 60–80% confluence and incubated for 2 h, following which 1 µg/mL LPS was added for a further 24-h incubation period. Conditioned media were collected and subjected to IL-6 determination using a mouse IL-6 ELISA kit purchased from Invitrogen (Carlsbad, CA, USA).

### 4.5. 12-O-Tetradecanoylphorbol Acetate (TPA)-Induced Mouse Ear Edema

Sixteen male and 16 female 8-week-old Balb/c mice were purchased from LASCO Biotechnology Co., Ltd. (Taipei, Taiwan) and housed in individual ventilated cages (IVCs) at 25 ± 1 °C, under a relative humidity maintained at 55 ± 5% and a 12:12-h light:dark cycle. The mice were randomly divided into four groups: control without treatment; 12-O-tetradecanoylphorbol acetate (TPA) treatment only; indomethacin followed by TPA treatment; and KXRG followed by TPA treatment. Thirty minutes after KXRG (0.5 mg/ear dissolved in PBS) or indomethacin (0.5 mg/ear dissolved in 50% ethanol) application, ear inflammation edema was induced by topical application of 2 μg TPA dissolved in 20 μL of acetone to both the inner and outer ear surfaces. At various durations post-TPA application, the ear thickness was measured using a spring-loaded micrometer (Dial Thickness Gauge, Peacock, Tokyo, Japan).

### 4.6. LPS-Induced Systemic Inflammation in Mice

Thirty-two male 8-week-old C57BL/6 mice were purchased from LASCO Biotechnology Co., Ltd. and kept in a housing environment as described above in 4.5. The mice were randomly divided into four groups: group 1, control; group 2, LPS injection at 5 mg/kg; group 3, LPS injection and fed with 25 mg/kg KXRG by oral gavage needle; group 4, LPS injection and fed with 50 mg/kg KXRG. After one week of adaptation, the mice were fed KXRG daily for 10 days (day 1 to day 10) and received an LPS intraperitoneal injection on day 5 to day 7. Serum collection and the Y-maze test were performed on day 11. Organs—including the duodenum, jejunum, ileum, kidneys, liver, hippocampus, and cerebral cortex—were collected and preserved in formalin for histologic examination and placed into liquid nitrogen for protein extraction.

### 4.7. Histopathological Analysis

Different organs were fixed in 4% formalin fixative, embedded in paraffin, sectioned into 4-μm-thick sections, and then stained with hematoxylin-eosin (HE) solution. Histological alteration and the degree of injury to renal and liver tissues were scored from 0–4, 0 points representing normal tissue, 1 point 1–10% abnormal, 2 points 11–25% abnormal, 3 points 26–45% abnormal, and 4 points greater than 46% abnormal. The small intestine was divided into the duodenum, jejunum, and ileum, and the villous height and crypt depth were measured. For each sample, at least 10 independent fields were observed by co-author Dr. Chin-Dong Chang, who is also an experienced pathologist.

### 4.8. Biochemical Analysis

The liver TAC and serum SOD were determined using a commercial ELISA kit (Biovision). The levels of proinflammatory cytokines IL-6, IL-1β, and TNF-α in the serum and cell culture conditioned medium were determined using kits purchased from Invitrogen. The assay protocol was according to the manufacturer’s instructions.

### 4.9. Y-Maze Analysis

The Y-maze consists of three arms, labeled A, B, and C. At the start of the test, the C arm was closed off, and the animal was allowed to explore the start of arms A and B for 5 min, then placed back into the cage and allowed to rest for 3 min. The maze was wiped with alcohol to remove any smell. Then, all three ends of the maze arms were opened, and the mouse was allowed to explore freely for 10 min, during which the number of times and the times at which the animal entered the three arms, in addition to the speed of movement, were recorded [17]. Spontaneous alteration behavior was defined as (consecutive entries into three different arms/total number of arm entries − 2) × 100; a higher percentage showed better memory and cognition [18]. Next, to evaluate the effects of KXRG on short-term spatial memory, with all three arms open, a food reward was placed at the end of arm C, and the animal was placed into arm A. After three sessions of training to enable the animal to recognize that the food was always placed in arm C, the mouse was returned to the home cage. After one day of rest and 8 h of starvation, the spatial memory of the animal was then tested in terms of its ability to remember that the food was located in arm C. The search for food was conducted five times, within a duration of 10 min. A digital camera was positioned above and software employed for analysis.

### 4.10. Western Blotting Analysis

Certain collected tissues were homogenized in RIPA buffer (50 mM Tris HCl pH 7.4, 50 mM NaCl, 5 mM EDTA, 1% NP-40, 1% sodium deoxycholate, 0.1% SDS, 1% aprotinin, 50 mM NaF, 0.1 mM Na_3_VO_4_) then centrifuged to collect cell lysate supernatants. The procedures of SDS-PAGE and protein transfer, and the antibodies used, were as described in our earlier report [48]. Digital images were obtained using a luminescence reader (Biospectrum-UVP, Cambridge, UK) and densitometry analysis was performed using UVP VisionWorks LS software (UVP, Cambridge, UK).

### 4.11. Statistics

Data analysis was performed using SPSS software version 20 (IBM, Armonk, NY, USA) provided by our university. The data are expressed as the mean ±SEM. Tukey post-hoc analysis was performed following one-way ANOVA, with *p* < 0.05 indicating statistical significance.

## 5. Conclusions

This study represents the first in which a flavonoid compound with significant anti-inflammatory activity was prepared from low-cost raw materials. The results of the present study indicated that the flavonoid triglycoside extracted from *C. brevistyla* seed dregs exerted in vivo anti-inflammatory effects.

KXRG has significant effects on both local and systemic inflammatory responses, at least in part through selectively targeting COX-2 and IL-6; it prevents LPS-induced tissue damage in the small intestine, liver, and kidneys, and reduces memory impairments and neuroinflammatory responses. Further studies are required to evaluate the safety of long-term use of KXRG to inform its potential development as a new drug to prevent and treat diseases associated with inflammation.

## Data Availability

The authors confirm that all data generated or analyzed during the present study are included in this published article.

## Supplementary Materials

The following are available online at https://www.mdpi.com/article/10.3390/molecules27072055/s1, Figure S1. 1H-NMR spectrum of KXRG in CD3OD; Figure S2. 13C-NMR spectrum and DEPT of KXRG in CD3OD; Figure S3. IR spectrum of KXRG; Figure S4. Mass spectrum of KXRG; Figure S5. HPLC chromatogram of KXRG; Table S1. The purity calculation of KXRG.

## Sample Availability

Samples of the compounds are not available from the authors.
