# Supplementary material for: Tea Seed Kaempferol Triglycoside Attenuates LPS-Induced Systemic Inflammation and Ameliorates Cognitive Impairments in a Mouse Model
Source: Molecules. 2022 Mar 22;27(7):2055. doi: 10.3390/molecules27072055 (PMC9000603; doi:10.3390/molecules27072055)
Supplement: Supplementary file 1 [file molecules-27-02055-s001.zip › molecules-1596805-supplementary.pdf]

SUPPLEMENTARY MATERIAL

Tea Seed Kaempferol Triglycoside Attenuates LPS-Induced Systemic Inflammation and Ameliorates Cognitive Impairments in a Mouse Model

Tsung-Ming Yeh [1,2,3], Ching-Dong Chang [1], Shyh-Shyan Liu [1], Chi-I Chang [2,*] and Wen-Ling Shih [2,*]

[1] Department of Veterinary Medicine, National Pingtung University of Science and Technology, Pingtung 912, Taiwan; ytm@mail.npust.edu.tw (T.-M.Y.); cdchang@mail.npust.edu.tw (C.-D.C.); lad@mail.npust.edu.tw (S.-S.L.)
[2] Department of Biological Science and Technology, National Pingtung University of Science and Technology, Pingtung 912, Taiwan
[3] General Research Service Center, National Pingtung University of Science and Technology, Pingtung 912, Taiwan
* Correspondence: changchii@mail.npust.edu.tw (C.-I.C.); wlshih@mail.npust.edu.tw (W.-L.S.); Tel.: +886-8-7703202 (ext. 5185) (C.-I.C.); +886-8-7703202 (ext. 5192) (W.-L.S.)

CONTENT

Figure S1. ^{1}H-NMR spectrum of KXRG in CD_3OD.
Figure S2. ^{13}C-NMR spectrum and DEPT of KXRG in CD_3OD.
Figure S3. IR spectrum of KXRG.
Figure S4. Mass spectrum of KXRG.
Figure S5. HPLC chromatogram of KXRG.
Table S1. The purity calculation of KXRG.

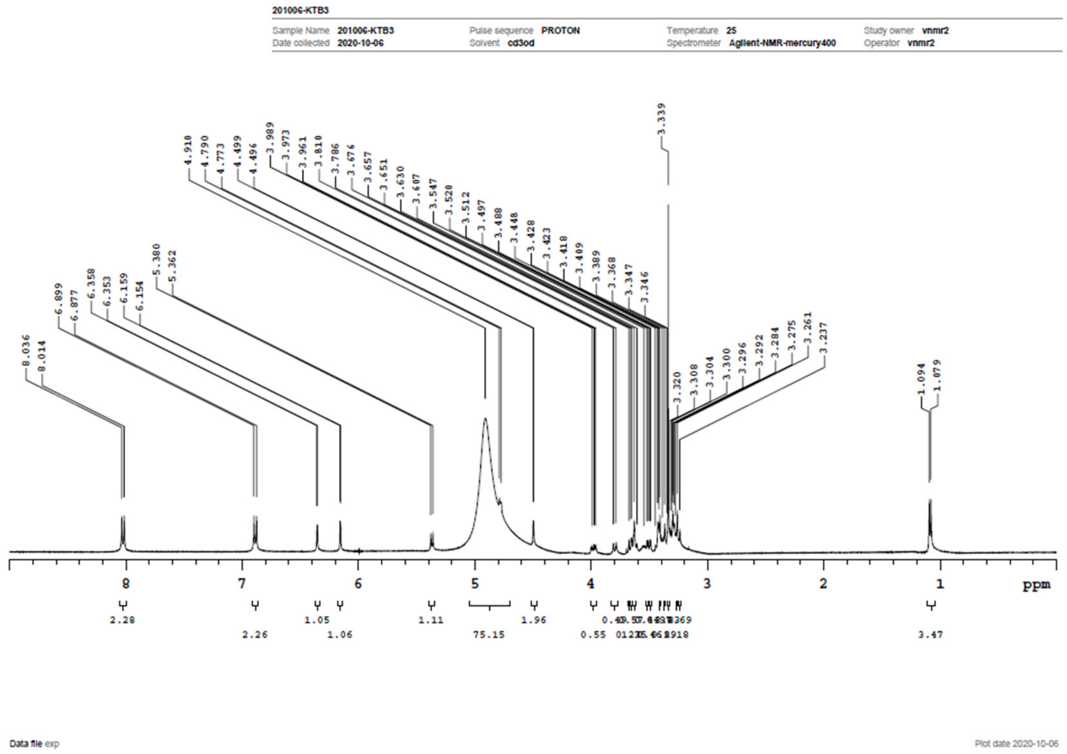

Figure S1. ^{1}H-NMR spectrum of KXRG in CD_3OD.

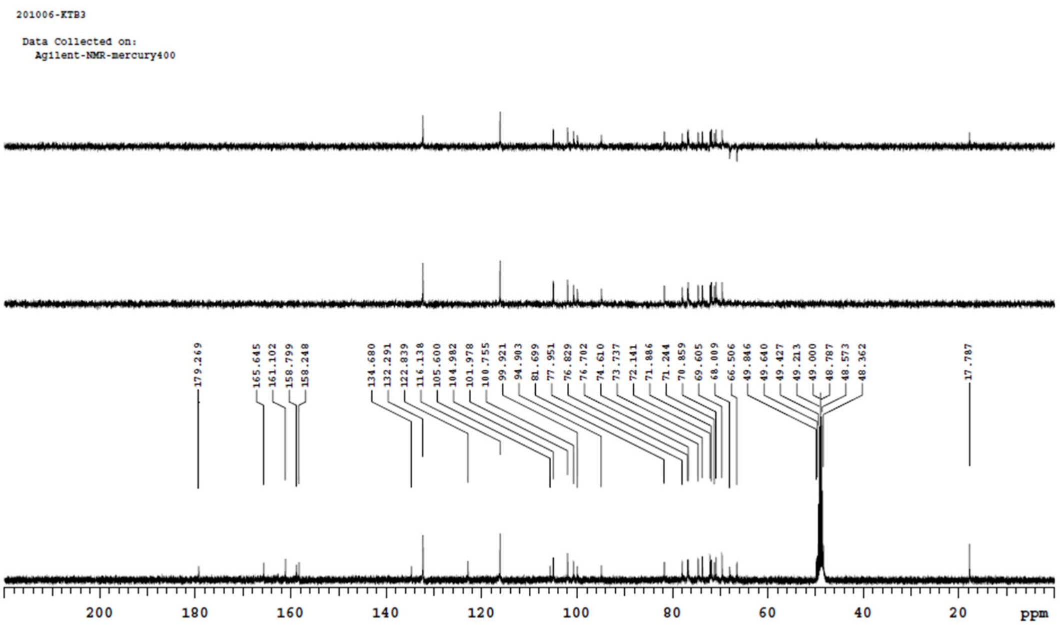

Figure S2. ^{13}C-NMR spectrum and DEPT of KXRG in CD_3OD.

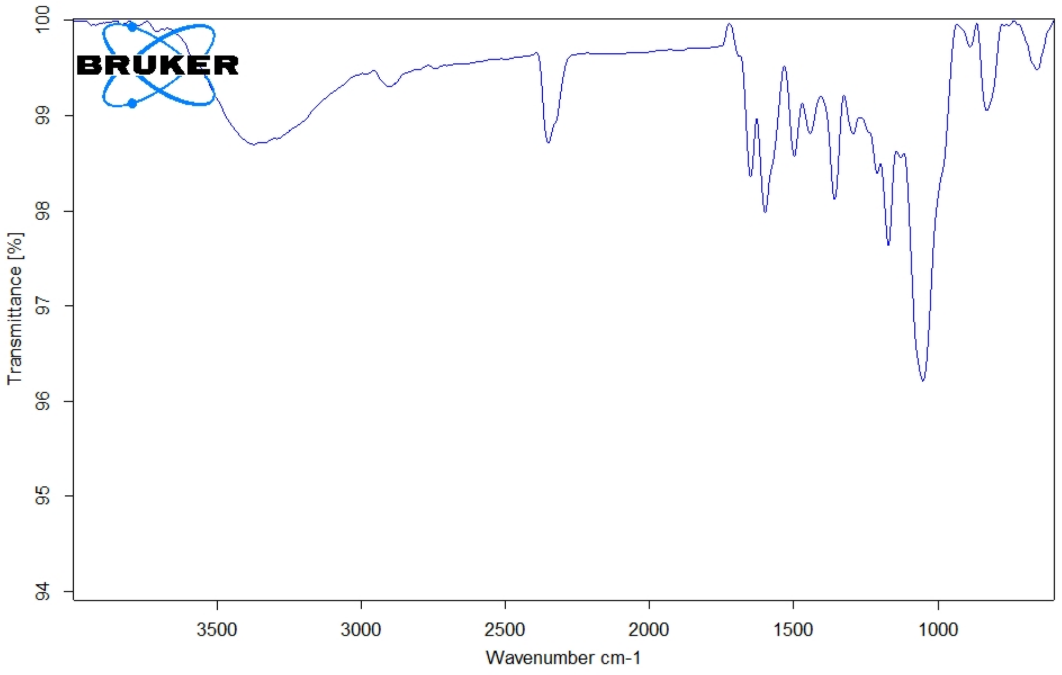

Figure S3. IR spectrum of KXRG.

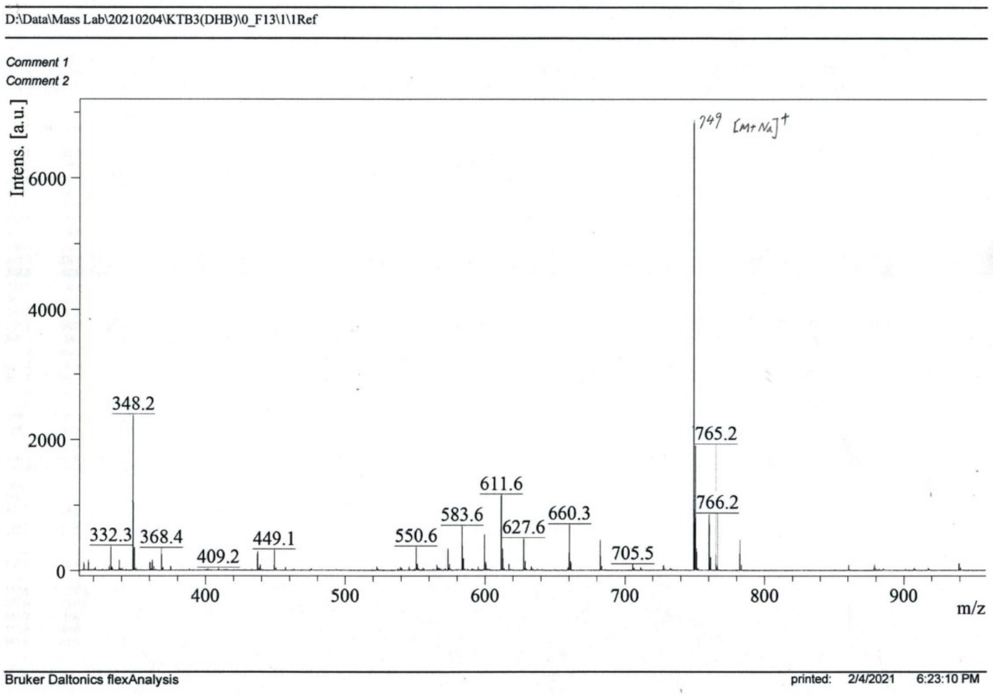

Figure S4. Mass spectrum of KXRG.

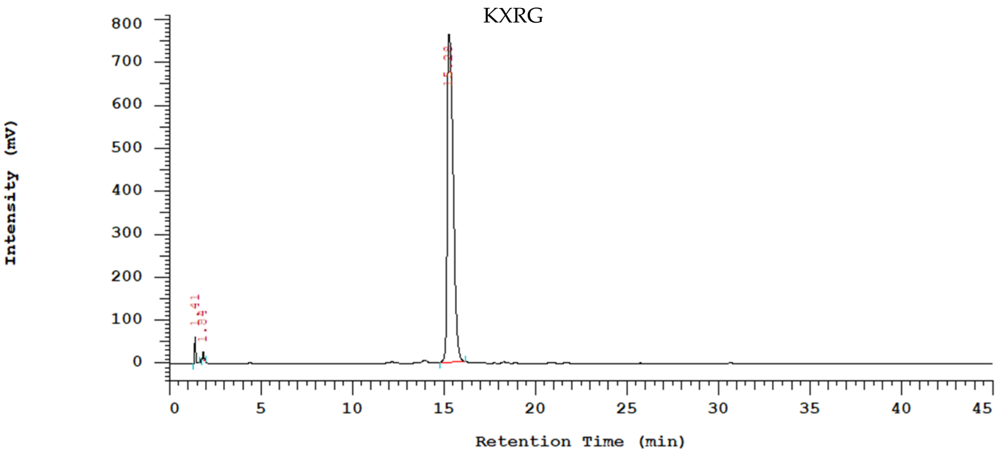

Figure S5. HPLC chromatogram of KXRG (detection wavelength, 280 nm).

Table S1. The purity calculation of KXRG.

Peak No.	Retention time	Area	Percentage (%)
1	1.41	0	0 (solvent peak)
2	1.84	110623	0.647
3	15.28	16999015	99.353
Total		17109638	100
